# Structural evaluation of sugar cane bagasse steam pretreated in the presence of CO_2_ and SO_2_

**DOI:** 10.1186/1754-6834-5-36

**Published:** 2012-05-22

**Authors:** Roberta Cristina Novaes Reis Corrales, Fabiana Magalhães Teixeira Mendes, Clarissa Cruz Perrone, Celso Sant’Anna, Wanderley de Souza, Yuri Abud, Elba Pinto da Silva Bon, Viridiana Ferreira-Leitão

**Affiliations:** 1 National Institute of Techonology, Ministry of Science and Techonology, Av. Venezuela, 82, sala 302, CEP 20081-312, Rio de Janeiro - RJ, Brazil; 2 National Institute of Metrology, Standardization and Industrial Quality, Av. Nossa Senhora das Graças, 50 – Xerém, CEP 25250-020, Duque de Caxias - RJ, Brazil; 3 National Institute of Science and Technology in Structural Biology and Bioimagens, Federal University of Rio de Janeiro, Av. Pedro Calmon, 550, Prédio da Reitoria - sala 801, Ilha do Fundão - CEP 21941-901, Rio de Janeiro – RJ, Brazil; 4 Department of Biochemistry, Institute of Chemistry, Federal University of Rio de Janeiro, Av. Athos da Silveira Ramos, 149, bloco A, Ilha do Fundão, CEP: 21941-909, Rio de Janeiro - RJ, Brazil

**Keywords:** Sugar cane bagasse, CO_2_ and SO_2_ steam pretreatment, SEM and TEM microscopy, XRD and FTIR spectroscopy

## Abstract

**Background:**

Previous studies on the use of SO_2_ and CO_2_ as impregnating agent for sugar cane bagasse steam treatment showed comparative and promising results concerning the cellulose enzymatic hydrolysis and the low formation of the inhibitors furfural and hydroxymethylfurfural for the use of CO_2_ at 205°C/15 min or SO_2_ at 190°C/5 min. In the present study sugar cane bagasse materials pretreated as aforementioned were analyzed by scanning and transmission electron microscopy (SEM and TEM), X-Ray Diffraction (XRD) and Infrared (FTIR spectroscopy) aiming a better understanding of the structural and chemical changes undergone by the pretreated materials.

**Results:**

SEM and TEM data showed that the structural modifications undergone by the pretreatment with CO_2_ were less pronounced in comparison to that using SO_2,_ which can be directly related to the combined severity of each pretreatment. According to XRD data, untreated bagasse showed, as expected, a lower crystallinity index (CI = 48.0%) when compared to pretreated samples with SO_2_ (CI = 65.5%) or CO_2_ (CI = 56.4%), due to the hemicellulose removal of 68.3% and 40.5%, respectively. FTIR spectroscopy supported SEM, TEM and XRD results, revealing a more extensive action of SO_2_.

**Conclusions:**

The SEM, TEM, XRD and FTIR spectroscopy techniques used in this work contributed to structural and chemical analysis of the untreated and pretreated bagasse. The images from SEM and TEM can be related to the severity of SO_2_ pretreatment, which is almost twice higher. The crystallinity index values obtained from XRD showed that pretreated materials have higher values when compared with untreated material, due to the partial removal of hemicellulose after pretreatment. FTIR spectroscopy supported SEM, TEM and XRD results. CO_2_ can actually be used as impregnating agent for steam pretreatment, although the present study confirmed a more extensive action of SO_2_.

## Background

There is a growing urgency to develop novel bio-based products and other innovative technologies that can overcome the widespread dependence on fossil fuels [1]. Unlike gasoline, ethanol is a renewable energy source produced through fermentation of sugar. In Brazil, ethanol is produced largely from sugar cane juice, known as first generation (1G) ethanol. The residual lignocellulosic biomass from the 1G ethanol industry (sugar cane bagasse and leaves) is, presently, for a collection of reasons, the most promising resource for the production of lignocellulosic (2G) ethanol [2]. However, although the sugar-ethanol industry generates bagasse in large quantities during the process of extraction of the sugar cane juice it is mostly used for co-generation, accounting for approximately 3% of the electricity available in Brazil [3].

Lignocellulosic biomass is mainly composed of cellulose, hemicellulose and lignin. The predominant component of lignocellulosic biomass is cellulose, a linear β (1,4)-linked chain of glucose molecules. It is non-toxic, renewable, biodegradable, modifiable and has great potential as an excellent industrial material [4,5]. The elementary fibrils are composed of crystalline and amorphous regions. Hemicelluloses are made up of C5 and C6 sugar, such as xylose, arabinose, galactose, glucose and mannose. Lignin accounts for about one fourth of the lignocellulosic biomass and is the third most abundant biopolymer only after cellulose and hemicellulose.

According to Fengel and Wegener [6], four elementary fibrils of cellulose are held together by a monolayer of hemicellulose, which generate 25 nm wide thread-like structures that are enclosed in a matrix of hemicellulose and lignin (associated with each other through physical interactions and covalent bonds).

The main steps for ethanol production from lignocellulosic biomass are pretreatment, hydrolysis, fermentation and distillation/purification. The pretreatment should enhance the fiber accessibility and consequently facilitate the subsequent steps of enzymatic hydrolysis and fermentation [7].

The raw material pretreatment step could represent up to 20% of the total costs of cellulosic ethanol production [8]. According to Galbe and Zacchi [9], an effective pretreatment should (a) improve cellulose digestibility; (b) produce low concentrations of degradation products derived from sugars and lignin; and (c) have a low energy demand.

Previous studies on steam pretreatment of bagasse employed CO_2_ as impregnating agent to replace the traditionally used SO_2_[10]. The use of CO_2_ was previously investigated in order to explore some advantages of this gas over SO_2_, such as high availability in the first-generation ethanol plants, low toxicity, low corrosivity and low occupational risk [10,11]. Although the use of CO_2_ provided equivalent results in comparison to those obtained when SO_2_ was used as impregnating agent, higher temperatures or longer times were necessary. Comparative results concerning glucose release and inhibitors formation (furfural and hydroxymethylfurfural – HMF) from steam pretreated bagasse were obtained under the conditions: 205°C/15 min using CO_2_ or 190°C/5 min using SO_2_. As previously reported by authors [10], the use of SO_2_ resulted in 79.7% of glucose after enzymatic hydrolysis and provided the formation of 0.80 g/100g of furfural and 0.18 g/100g of HMF (dry bagasse). When CO_2_ was employed, the yield of glucose reached 86.6% and the values for furfural (0.9 g/100g) and HMF (0.2 g/100g) were very similar to those reported for SO_2._

FTIR spectroscopy and electron microscopy have been used for the analysis of structural and morphological modifications in the biomass after pretreatment [12-14]. The present work evaluated structural and chemical changes of SO_2_ and CO_2_ steam pretreated sugar cane bagasse in comparison to the untreated material using electron microscopy, X-ray diffraction (XRD) and infrared spectroscopy (FTIR).

## Results and discussion

### Scanning electron microscopy (SEM) and transmission electron microcopy (TEM)

**Figure 1:**
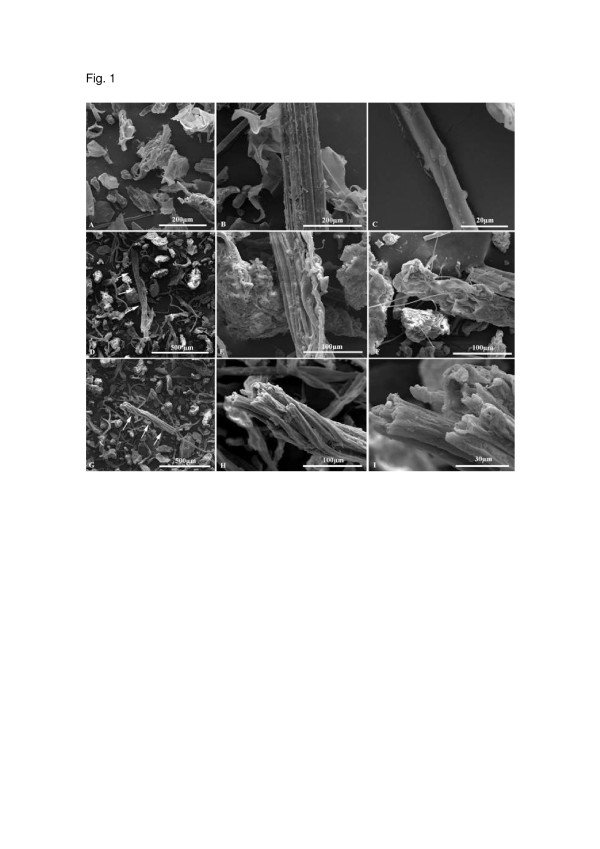
**SEM images of untreated sugar cane bagasse (A, B and C)**; **(A)** General view of the sample showing the fibers (mainly); (**B** and **C**) higher magnification image of fiber surface; SEM images of sugar cane bagasse pretreated with SO_2_ (190°C/5 min) (**D**, **E** and **F**); (**D**) General view of the sample showing the fibers (mainly); (**E**) higher magnification image of fiber surface (arrows in the **D** image); (**F**) higher magnification image of fiber surface extremity; SEM images of the bagasse pretreated with CO_2_ (205°C/15 min) (**G, H** and **I**); (**G**) General view of the sample showing the fibers (mainly); (**H**) and (**I**) higher magnification images of fiber surface (arrows in the **G** image).

The use of scanning electron microscopy as an analytical technique proved to be of great importance and versatility for studying the biomass structure. Figure 1 shows the morphological characteristics of the steam pretreated bagasse in the presence of CO_2_ or SO_2_ as well as of the untreated material, obtained by scanning electron microscopy (SEM).

Untreated bagasse sample (Figure 1A, B, C) presents a rigid and compact morphology, while the ones submitted to pretreatment with SO_2_ (Figure 1D, E, F) or CO_2_ (Figure 1G, H, I) exhibited a more disorganized morphology, with greater exposure of the fibers.

After pretreatment, the most exposed cell wall structure allows for a greater accessibility to hydrolytic enzymes, which facilitates the hydrolysis of lignocellulosic biomass.

**Figure 2:**
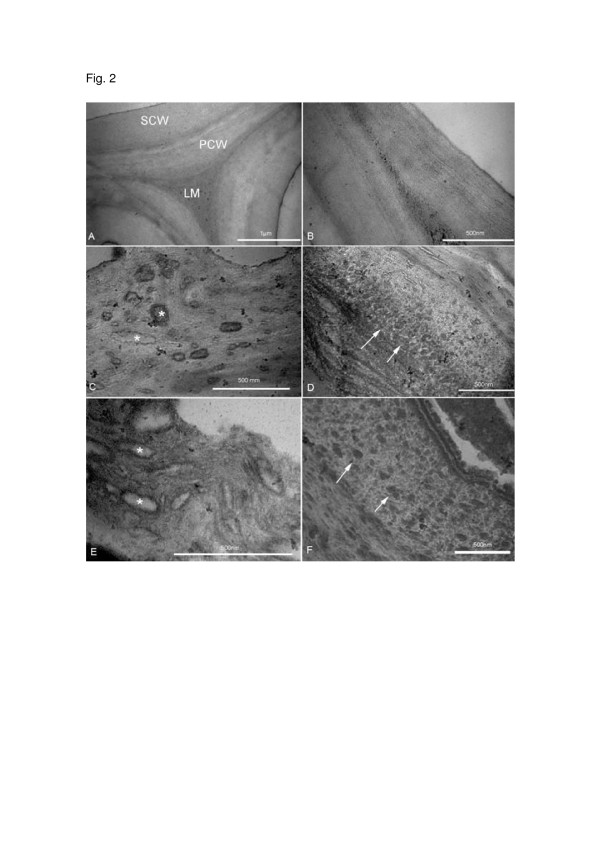
**TEM images of untreated bagasse showing the following cell wall layers**: primary cell wall (PCW), secondary cell wall (SCW) and middle lamella (ML) (Fig. **A**). Fig. **B** – higher magnification of SCW showing the cellulose microfibrils orientation. Fig. **E, F** – SO_2_ (190°C/5 min) pretreated cell wall. Large pores with distinct size and shape are observed (Fig. **C**, asterisks). Compaction of cell wall matrix was visualized forming round and elongated structures (Fig. **D**, arrows). Fig. **C, D** – CO_2_ (205°C/15 min) pretreated cell wall. After treatment pores (Fig. **E**, asterisks) and structures formed by compaction of cell wall matrix (Fig. **F**, arrows) are seen spread at the outer region of secondary cell wall

Transmission electron microscopy (TEM) has been used as a suitable method to determine the effect of pretreatment within the plant cell wall [15]. TEM images of untreated sugar cane bagasse clearly showed that the primary cell wall (PCW), secondary cell wall (SCW) and middle lamella (ML) were well preserved (Figure 2A, B). These structures were bonded strongly together giving rise to a typical highly compact architecture of cell walls. As it is a thicker and more rigid structure in the bagasse, the SCW, where cellulose microfibrils are arranged in parallel position, is responsible for cell wall integrity (Figure 2)B. The pretreated CO_2_ samples show, in the cell wall, large pores with different size and shape (Figure 2C, D). Remarkably, most of the pores were formed in the outer region of the cell wall. When SO_2_ was used as impregnating agent, the secondary cell wall, especially the outer region, was also severely disrupted leading to the appearance of large irregular shaped pores (Figure 2E, F) as a result of partial solubilization of ultrastructural cell wall components. Similar results were recently reported by Chundawat and co-workers [15] in corn stover after ammonia fiber expansion (AFEX) treatment.

The AFEX pretreatment strategy revealed that cellulose hydrolysis increased roughly five-fold when compared to untreated samples. In addition, when both CO_2_ and SO_2_ were employed, coalescent particles with round or elongated shapes were found in the cell wall (Figure 2D, F). They seem to be formed by the process of coalescence of cell wall matrix components (hemicelluloses and lignin). The change in cell wall morphology observed when bagasse was pretreated with either CO_2_ or SO_2_ result in the increase of the cell wall porosity. At nanoscale, the limited cell wall matrix porosity is considered an important factor that impairs cellulase penetration and accessibility to cellulose fibrils, therefore, contributing to biomass recalcitrance [16,17].

**Figure 3:**
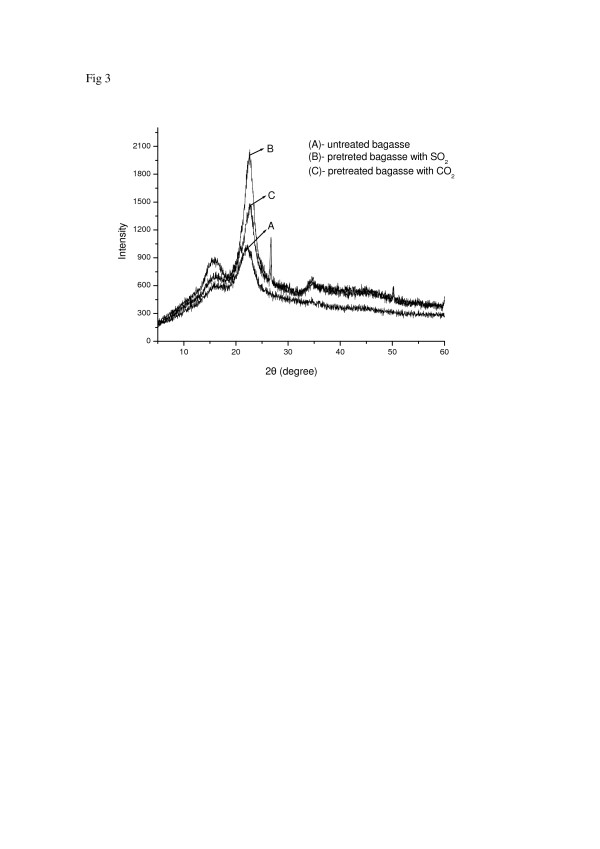
**Diffractograms of the sugar cane bagasse samples**: (A) untreated bagasse; (B) pretreated bagasse with SO_2_ (190°C/5 min); (C) pretreated bagasse with CO_2_ (205°C/15 min)

Data from SEM and TEM showed that both pretreatments were effective with respect to structural changes, increasing the surface exposure of the bagasse samples. However, the morphology of CO_2_ pretreated material is more preserved than that of the SO_2_ material. This result can be directly related to the higher combined severity for the pretreatment using SO_2_ (1.7), when compared to combined severity of the pretreatment with CO_2_ (0.9) as impregnating agent. It is important to emphasize that the combined severity factor [18] takes into account besides time and temperature of the steam pretreatment, the acidity generated in the reaction media by the formation of sulfuric and carbonic acid and the release of organic acids, such as acetic acid from the raw material, as indicated by the pH drop after pretreatment (pH 1.7 (SO_2_); pH 3.8 (CO_2_)) [10].

### X-ray diffraction (XRD)

Figure 3 shows diffractograms of untreated bagasse (A) and sugar cane bagasse pretreated with SO_2_ (B) or CO_2_ (C). As can be observed, all samples exhibit typical cellulose diffraction peaks, where the highest peak corresponds to the 002 crystallographic planes. The crystallinity index was calculated according to Equation 1 (Methods session). The untreated sugar cane bagasse showed a lower crystallinity (CI = 48.0%) when compared to samples pretreated with SO_2_ (CI = 65.5%) and CO_2_ (CI = 56.4%). Many studies indicate that there is an increase in the value of this index when the biomass is subjected to pretreatment by steam explosion [19]. The phenomenon is due mainly to the removal of a certain amount of lignin and hemicellulose (amorphous substances) and not necessarily due to changes in the crystalline structure of the biomass.

As expected the crystallinity index for the bagasse pretreated with SO_2_ (65.5%) was higher than that of CO_2_ pretreated bagasse (56.4%). Indeed, it was observed a more effective removal of hemicellulose to the liquid fraction (63.8%: 7.0% xylose as oligomers and 56.8% of monomeric xylose) using the SO_2_ steam pretreatment than that using CO_2_ that showed 40.5% hemicellulose removal : (21.5%) of xylose in oligomeric form and 19.0% in monomeric form) [10]. In this work, the increase of the crystallinity index in the pretreated samples is explained by the partial removal of hemicellulose fraction. The amount of glucose released from cellulose (amorphous region) was not relevant in the pretreatment step.

The high-pressure steam modifies the plant cell wall structure, yielding a dark brown material from which partially hydrolyzed hemicelluloses are easily recovered by water-washing, leaving a water-insoluble fraction composed of cellulose, residual hemicelluloses and a chemically modified lignin [20].

### FTIR spectra

**Figure 4:**
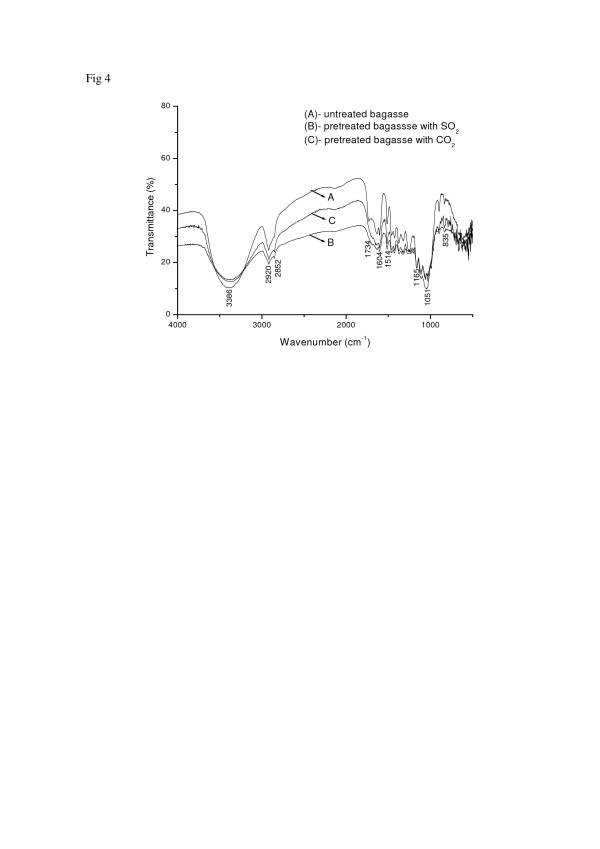
**FT-IR spectra**: (A) untreated sugar cane bagasse; (B) pretreated bagasse with SO_2_ (190°C/5 min); (C) pretreated bagasse with CO_2_ (205°C/15 min)

In order to understand the changes in the chemical structure after pretreatment, infrared spectra (Figure 4) of the untreated sample (A) and pretreated samples (B and C) were obtained. The assignments given to the absorption bands were referred to the collection of literature Table 1 [21-26].

The band at 1514 cm^-1^ has been chosen as an internal standard, since this band is present in all spectra and it is well defined.

The main features of these spectra are attributed to the presence of lignin, hemicellulose and cellulose; the natural components of lignocellulose fibers. Infrared spectra of pretreated samples are similar to the untreated ones, which show that the pretreatment conditions did not promote drastic changes in the chemical structure. The values in Tables 1, 2, 3 and 4 represent the relative absorbance of main functional groups stretching (O-H, C-Ph, C=C, OCH_3_, C=O).

**Table 1:** Relative intensity of bands in the infrared spectrum of different groups in the untreated and pretreated sugar cane bagasse samples

**Assignment of FT-IR absorption of sugar cane bagasse**	**Relative absorbance of different groups in bagasse samples**			
	**Maximum band position (cm**^**-1**^**)**	**Untreated bagasse**	**Pretreated bagasse with SO**_**2**_	**Pretreated bagasse with CO**_**2**_
O-H stretching (H-bonded)	3386	0.30	0.51	0.50
O-H vibration of phenolic group	1375	0.79	0.93	0.90
O-H stretching of secondary alcohol	1165	0.51	0.71	0.65
O-H stretching of primary alcohol	1051	0.29	0.61	0.57
C-O-C stretching	1110	0.40	0.65	0.58
C-O stretching of phenols	1250	0.69	0.95	0.89
C-H aliphatic axial deformation	2920	0.71	0.78	0.78
C-H aliphatic angular deformation	1427	0.85	0.94	0.91
C-H vibration of methoxyl group	2852	0.85	0.87	0.89
C-H angular deformation of methoxyl group	1462	0.88	0.95	0.93
C-Ph vibration	1604	0.88	0.97	0.94
C=C aromatic skeletal vibration	1633	0.89	0.99	0.98
β-glycisidic linkages	897	1.14	1.24	1.19
C=O stretching	1735	1.03	1.19	1.24

**Table 2:** **Relative absorbance of OCH**_**3**_**group (cm**^**-1**^**) according to the FTIR spectrum of the sugar cane bagasse**

**OCH**_**3**_**group bands (cm**^**-1**^**)**	**Untreated bagasse**	**Pretreated bagasse with SO**_**2**_	**Pretreated bagasse with CO**_**2**_
2852	0.85	0.87	0.89
1462	0.88	0.95	0.93
1427	0.85	0.94	0.91
Mean value	0.86	0.92	0.91

**Table 3:** **Relative absorbance of O-H group (cm**^**-1**^**) according to the FTIR spectrum of the sugar cane bagasse**

**OH group bands (cm**^**-1**^**)**	**Untreated Bagasse**	**Pretreated bagasse with SO**_**2**_	**Pretreated bagasse with CO**_**2**_
3386	0.3	0.51	0.50
1375	0.79	0.93	0.90
1165	0.51	0.71	0.65
1051	0.29	0.61	0.57
Mean value	0.47	0.69	0.66

**Table 4:** **Relative absorbance of aromatic ring (cm**^**-1**^**) according to the FTIR spectrum of the sugar cane bagasse**

**Aromatic ring bands (cm**^**-1**^**)**	**Untreated Bagasse**	**Pretreated bagasse with SO**_**2**_	**Pretreated bagasse with CO**_**2**_
1604	0.88	0.97	0.94
1633	0.89	0.99	0.98
Mean value	0.88	0.98	0.96

The absorption at 2920 cm^-1^ could be attributed to C-H aliphatic axial deformation in CH_2_ and CH_3_ groups from cellulose, lignin and hemicellulose.

Usually the absorptions of O-H stretching occur in 3100–3600 cm^-1^ range. The band observed at 3386 cm^-1^ seems to be characteristic of OH groups present in lignin and carbohydrates. From Table 3, it could be observed that this band has higher values of relative absorbance in the case of pretreated samples when compared to untreated one. This result could be attributed to the chemical changes observed when sugar cane bagasse is pretreated with SO_2_ or CO_2_.

The relative absorbance of the bands of primary and secondary OH groups at 1051 cm^-1^ and 1165 cm^-1^ of untreated sugar cane bagasse is lower than the pretreated ones (Tables 1 and 3). It is worth noting that the relative absorbance of pretreated bagasse with SO_2_ is even higher than the one pretreated with CO_2_. This could be explained by the fact that SO_2_ provides a lower pH and consequently a higher combined severity, which resulted in a more exposed structure.

According to Nada and co-workers [13], the band at 2852 cm^-1^ is assignable to vibration of OCH_3_ groups, which is commonly present in lignin (Table 2). This OCH_3_ group could also be attributed to acetyl from hemicellulose. It could be observed that only a slight increase of 6.5% and 5.5% of the relative absorbance of OCH_3_ groups after pretreatment using SO_2_ or CO_2_, respectively, as expected after an efficient pretreatment process.

When comparing the relative absorbance of the OCH_3_ group and OH group (Tables 2 and 3), it is possible to note that there was a significant increase in the relative absorbance after pretreatment of OH groups (31.9% and 28.8%, using SO_2_ and CO_2_ in the pretreatment, respectively), while this is not observed for the OCH_3_ groups (6.5% and 5.5%, using SO_2_ and CO_2_, respectively). This data indicate the conversion of lignin methoxyl groups into phenolic groups during pretreatment [25].

The bands at 1604 cm^-1^ and 1633 cm^-1^ are attributed to C-Ph and C=C, respectively. These bands are generally found in the lignin aromatic structure. The relative absorbance of these vibrations are higher (around 9.3%) after pretreatment (Table 4), which confirms the conversion of lignin methoxyl groups into phenolic groups.

The band at 1735 cm^-1^ is referred to the acetyl groups present in the hemicellulose. The pretreated bagasse with CO_2_ or SO_2_ exhibits a higher relative absorbance when compared to untreated sample 16.9% and 15.5%, respectively (Table 1). The former results can be explained by the amount of hemicelullose fraction removed in each pretreatment, 40.5% and 63.7% for using CO_2_ and SO_2_, respectively [10].

The signal at 897 cm^-1^ is attributed to β-glycosidic linkages between monosaccharide units and it is also higher for the pretreated samples (8.8% and 4.4% with SO_2_ and CO_2_, respectively), as expect after bagasse fibers exposure.

## Conclusions

The analysis by SEM, TEM, XRD and FTIR spectroscopy of steam pretreated bagasse in the presence of CO_2_ at 205°C/15 min or SO_2_ at 190°C/5 min showed significant differences amongst the untreated and the pretreated materials. It was observed in the outer region of the cell wall (SCW), upon pretreatment, the formation of large pores with different sizes and shapes which were more prominent when SO_2_ was the impregnating agent. It was also observed in both cases the formation, in the cell wall, of coalescent particles with round or elongated shapes likely formed by lignin and/or hemicellulose.

The morphology of CO_2_-pretreated bagasse was more preserved than that of the SO_2_-preatrement, likely due to its lower combined severity factor of 0.9 in comparison to that of SO_2−_ pretreatment of 1.7. It was also observed that both impregnating agents, SO_2_ or CO_2_, behaved in a quite similar way.

The crystallinity index values obtained from XRD patterns showed that pretreated materials have higher values (CI (SO_2_) = 65.5%, CI (CO_2_) = 56.4%) when compared with untreated material (CI = 42.5%), due to the partial removal from the bagasse of its hemicelluloses content. The results of FTIR spectra also showed changes in the chemical structure of materials pretreated with CO_2_ and SO_2_, mainly in OCH_3_, OH and C=O groups; which supported the data from the XRD, SEM and TEM analysis.

## Methods

The use of SO_2_ and CO_2_ as an impregnating agent for sugar cane bagasse treatment was previously studied [10]. In the present study the most promising pretreated materials were selected for further studies on structural modifications. Bagasse samples steam pretreated in the presence of CO_2_ (205°C/15 min) and SO_2_ (190°C/5 min) and also untreated bagasse were submitted to the following techniques: electron microscopy, X-ray diffraction and infrared spectroscopy. All samples were sieved (< 1.8 mm) before analyses.

### Scanning electron microscopy (SEM)

Scanning electron microscopy (SEM - FEI / Inspect S50 model) was used to observe modifications on bagasse fibers. Samples were adhered to carbon tape and sputter coated with gold (sputter Emitech / K550 model) and observed in the SEM through the use of an acceleration voltage of 20 KV and working distance of around 38 mm. Hundreds of SEM images were obtained on different areas of the samples to guarantee the reproducibility of the results.

### Transmission electron microscopy (TEM)

Transmission electron microscopy (FEI Tecnai G2 12 Spirit) was used to observe the ultrastructural changes within the cell wall. Each condition (untreated material, pretreated with SO_2_ and CO_2_ material) was analyzed in triplicate. Each individual sample was studied by an unbiased random selection of fibers that represent the total population. Samples were dehydrated in an increasing acetone series and embedded in Spurr resin. Ultrathin sections, 70nm, were obtained in the LEICA ultramicrotome and deposited onto copper grids. The sections were stained with 5% uranyl acetate and lead citrate and observed in the TEM with an acceleration voltage of 120 kV.

### X-ray diffraction (XRD)

Crystallinity of the cellulose fibers was evaluated by X-ray diffraction by means of a Diffractometer MiniFlex – Rigaku and filtered copper Kα radiation (λ = 0,1542 nm) by a monochromator at 30 KV voltage and 15 mA electric current, with a speed of about 2 degrees per minute and scanning at an angle (2θ) in the range of 2-60. The crystallinity of lignocellulose biomass accounts for the relative amount of total crystalline cellulose in the solid component. The crystallinity is strongly influenced by the composition of biomass; the relative amount of lignin, hemicellulose and cellulose varies according to the nature of the biomass. The crystallinity index (CI) was obtained from the ratio between the intensity of the 002 peak (I_002_, 2θ = 22.5) and the minimum dip (I_am_, 2θ = 18.5) between the 002 and the 101 peaks according to Equation 1 [14,27].

(1) $$\%CI=\left[\left(I_{002}-I_{\mathrm{am}}\right)/I_{0}{}_{02}\right]\times 100$$

Where I_002_ is the intensity of plane 002 and I_am_ is related to the amorphous structure.

### Infrared spectroscopy (FTIR)

The infrared spectra (wave numbers in cm^-1^) were obtained on a Magma - IR 560 E.S.P – Nicolet spectrophotometer, by means of a KBr disk containing 3% finely ground samples. Thirty-two scans were taken of each sample recorded from 4000 to 400 cm^-1^ at a resolution of 4 cm^-1^. The relative absorbance values were obtained with four decimal units; however, only two decimal units were plotted in the data showed in Tables 1, 2, 3 and 4.

## Abbreviations

FTIR, Fourier transform infrared spectroscopy; SEM, Scanning electron microscopy; TEM, Transmission electron microscopy; XRD, X-Ray diffraction spectroscopy; CI, Crystallinity index.

## Competing interests

The authors declare that they have no competing interests.

## Authors’ contributions

VL and CP designed and carried out the experiments of pretreatment, the characterization of biomass, the discussion of results and revision of the manuscript. RC carried out SEM and XRD experiments and prepared the manuscript. FM carried out FTIR spectroscopy experiments and analyzed the results. CS and YA performed TEM analysis and discussed the results. EB and WS discussed the results and reviewed the manuscript. All authors read and approved the final version of the manuscript.
